# Delivery of antisense oligonucleotides for splice‐correction of androgen receptor pre‐mRNA in castration‐resistant prostate cancer models using cell‐penetrating peptides

**DOI:** 10.1002/pros.24309

**Published:** 2022-01-31

**Authors:** Maria V. Luna Velez, Omar Paulino da Silva Filho, Gerald W. Verhaegh, Onno van Hooij, Najoua El Boujnouni, Roland Brock, Jack A. Schalken

**Affiliations:** ^1^ Department of Urology, Radboud Institute for Molecular Life Sciences Radboud University Medical Center Nijmegen the Netherlands; ^2^ Department of Biochemistry, Radboud Institute for Molecular Life Sciences Radboud University Medical Center Nijmegen the Netherlands; ^3^ CAPES Foundation Ministry of Education of Brazil Brasília Brazil; ^4^ Department of Medical Biochemistry, College of Medicine and Medical Sciences Arabian Gulf University Kingdom of Bahrain

**Keywords:** antisense oligonucleotides, *AR‐V7*, castration‐resistant prostate cancer, cell‐penetrating peptide, PepFect 14

## Abstract

**Background:**

Cell‐penetrating peptides (CPPs) are a promising approach for delivering antisense oligonucleotides (AONs) as they form nanosized complexes through noncovalent interactions that show efficient cellular uptake. Previously, we have designed an AON system to correct splicing of the androgen receptor (*AR*) pre‐mRNA, thereby preventing the generation of the splice variant *AR‐V7* mRNA. AON‐mediated knockdown of *AR‐V7* resulted in inhibition of androgen‐independent cell proliferation. In this study, we evaluated the CPP‐mediated delivery of this AON into castration‐resistant prostate cancer cell line models 22Rv1, DuCaP (dura mater cancer of the prostate), and VCaP (vertebral cancer of the prostate).

**Methods:**

Nanoparticles (polyplexes) of AONs and CPPs were formed through rapid mixing. The impact of the peptide carrier, the formulation parameters, and cell incubation conditions on cellular uptake of fluorescently labeled AONs were assessed through flow cytometry. The cytotoxic activity of these formulations was measured using the CellTiter‐Glo cell viability assay. The effectivity of CPP‐mediated delivery of the splice‐correcting AON‐intronic splicing enhancer (ISE) targeting the ISE in the castration‐resistant prostate cancer (CRPC)‐derived 22Rv1, DuCaP, and VCaP cells was determined by measuring levels of *AR‐V7* mRNA normalized to those of the human heterochromatin protein 1 binding protein 3 (*HP1BP3*). Western blot analysis was used to confirm AR‐V7 downregulation at a protein level. The cellular distribution of fluorescently labeled AON delivered by a CPP or a transfection reagent was determined through confocal laser scanning microscopy.

**Results:**

The amphipathic and stearylated CPP PepFect 14 (PF14) showed higher uptake efficiency than arginine‐rich CPPs. Through adjustment of formulation parameters, concentration and incubation time, an optimal balance between carrier‐associated toxicity and delivery efficiency was found with a formulation consisting of an amino/phosphate ratio of 3, 0.35 μM AON concentration and 30 min incubation time of the cells with polyplexes. Cellular delivery of AON‐ISE directed against *AR* pre‐mRNA achieved significant downregulation of *AR‐V7* by 50%, 37%, and 59% for 22Rv1, DuCaP, and VCaP cells, respectively, and reduced androgen‐independent cell proliferation of DuCaP and VCaP cells.

**Conclusions:**

This proof‐of‐principle study constitutes the basis for further development of CPP‐mediated delivery of AONs for targeted therapy in prostate cancer.

## 1 INTRODUCTION

Patients with advanced prostate cancer have a 5‐year relative survival rate of 30%.^1^ Current therapy, directed to inhibit the androgen/androgen receptor signaling axis, only achieve modest survival benefits as the disease develops soon into a hormone‐refractory state known as castration‐resistant prostate cancer (CRPC). The elevated expression of C‐terminally truncated androgen receptor splice variants has been described as a mechanism of CRPC progression. Variants such as AR‐V7 can act as constitutively active transcription factors, promoting androgen‐independent tumor growth.^2^, ^3^ Previously, we designed two antisense oligonucleotides (AONs) to target splicing enhancers within the *AR* pre‐mRNA, responsible for the generation of an *AR‐V7* transcript. AON‐mediated inhibition of *AR‐V7* generation resensitized cells to androgen depletion, inducing apoptosis of diverse CRPC cell line models.^4^

Translation of AON‐based therapeutic approaches into an in vivo application requires additional tailoring to ensure specific cellular targeting, sufficient cellular uptake and endosomal release, and to prevent a rapid clearance by the body.^5^ Cationic cell‐penetrating peptides (CPPs) spontaneously associate with AONs into polyplexes that yield cellular uptake, both in vitro and in vivo.^6^, ^7^ The amphipathic CPP PepFect 14 (PF14) has shown superior activity in the cellular delivery of diverse oligonucleotides due to its capacity to induce endosomal release.^8^, ^9^

This study first demonstrates that PF14 outperforms the two stereoisomers of nona‐arginine and the human lactoferrin‐derived peptide (hLF)^10^, ^11^ concerning uptake efficiency. We next defined the optimal conditions to increase uptake efficiency while minimizing CPP‐associated toxicity. Polyplexes of PF14 and the splicing‐correcting AON‐intronic splicing enhancer (ISE) resulted in a significant downregulation of *AR‐V7* mRNA levels in three different CRPC cell lines, which was accompanied by a decrease in cell viability under castrate androgen conditions. These results demonstrate the feasibility of using CPPs such as PF14 to complement AON technology for targeting prostate cancer cells.

## 2 MATERIAL AND METHODS

### 2.1 Cell culture

The CRPC‐derived 22Rv1 (ATCC# CRL‐2505), DuCaP (dura mater cancer of the prostate), and VCaP (vertebral cancer of the prostate) (kindly provided by dr. Kenneth J. Pienta, Johns Hopkins) and the cervical adenocarcinoma HeLa cell line (ATCC: CCL‐2) were cultured in Roswell Park Memorial Institute (RPMI)−1640 medium (Invitrogen), supplemented with 2 mM l‐Glutamine and 10% fetal calf serum (FCS; Sigma‐Aldrich). Pancreatic carcinoma MIA‐PaCa‐2 cells (ATCC# CRL‐1420) were grown in Dulbecco's modified Eagle medium (DMEM) (Invitrogen) with 4.5 g/ml glucose and 1 mM pyruvate, supplemented with 2 mM l‐Glutamine and 10% FCS and 2.5% of Horse serum (Invitrogen). 22Rv1, DuCaP, and VCaP cell lines were authenticated in 2016 using the PowerPlex 21 system (Promega) by Eurofins Genomics. Cell cultures were maintained in a humidified atmosphere at 37°C and 5% CO_2_.

### 2.2 AONs

An RNA AON of 22 nucleotides (AON‐ISE) and a control sense oligonucleotide (SON‐ISE) were previously described.^4^ Both oligonucleotides were modified with a phosphorothioate backbone and 2′‐O‐methyl groups at the ribose (Biolegio). The oligonucleotides were dissolved in ultrapure water. The 5’ Cy3‐ or Alexa Fluor 568 (AF568)‐labeled AON Luc‐S‐oligo, previously described, were used for microscopy and flow cytometry.^12^

### 2.3 CPPs and polyplex formulations

The peptides PF14 (Stearyl‐AGYLLGKLL‐Orn‐Orn‐LAAAAL‐Orn‐Orn‐L‐l‐NH_2_, Orn corresponding to ornithine), hLF derived from human lactoferrin (Ac‐KCFQWQRNMRKVRGPPVSCIKR‐NH_2_) and nona‐arginine (L)‐R9 (Ac‐RRRRRRRRR‐NH_2_) and the d‐enantiomer (D)‐r9 (Ac‐rrrrrrrrr‐NH_2_) were purchased from EMC microcollections. The noncovalent peptide complexes (or polyplexes) of CPPs and AONs were formed based on the amino/phosphate (N/P) ratio, representing the ratio between the positively charged peptide side‐chain amino groups (N = nitrogen) and the negatively charged phosphorothioate (P) groups in the backbone of the AON. Polyplexes were generated by diluting the peptide and AON with ultrapure water at 10x their final concentration and pipetting equal volumes of both solutions simultaneously against the wall of a polypropylene 1.5 ml centrifuge tube. Before cell incubation, the polyplexes were incubated at room temperature for 1 h.

### 2.4 Dynamic light scattering

Size measurements of polyplexes were performed on a Zetasizer Nano S, using a HeNe laser with 4 mW, 633 nm. Polyplexes were diluted to a concentration of 0.2 µM with respect to the oligonucleotide in ultrapure water. For the determination of size, three technical replicates were performed per sample with an automatic selection for the number of runs. For size measurements, the backward scatter was used. The data analysis was carried out with ZetaSizer software 7.03.

### 2.5 Flow cytometry

To assess cellular uptake, 20,000 cells (VCaP) or 500 cells (MIA‐PaCa‐2 and HeLa) were cultured per well of a 96‐well culture plate 1 day before the experiment in a medium containing charcoal‐stripped (i.e., androgen‐free) serum (CSS). Polyplexes were formed with (L)‐R9, (D)‐r9, hLF and PF14 peptides and the Cy3‐labeled AON at an N/P ratio of 5, with 0.5 µM of the oligonucleotide, diluted in ultrapure water. Cells were incubated with polyplex mixes for 2 h at 37°C, washed and incubated for 96 h in CSS‐containing medium. For experiments with 30 min of cell incubation, polyplexes were formed at N/P ratios of 1, 3, and 5 with the final oligonucleotide concentration of 0.2, 0.35, and 0.5 µM. Incubation with naked AON was used to determine background uptake. X‐tremeGENE 9‐mediated transfection was performed according to the manufacturer's instructions (Roche). A mix of transfection reagent alone, that is, without AON, was used as nontransfected control. Four days after CPP treatment or transfection, cells were detached by trypsinization for 5 min, spun down and resuspended in 100 µl phosphate‐buffered saline (PBS). Fluorescence was measured with a fluorescence‐activated cell sorting‐Calibur flow cytometer, using a 15‐mW, 488 nm argon‐ion laser for excitation (BD Biosciences). Forward scatter‐height (FSC‐H) and median fluorescence intensity were analyzed using the FlowJo X software (FlowJo LLC). Results were based on > 5000 cells gated based on forward and side scatter.

### 2.6 Cell viability assay

To determine optimal conditions for PF14‐mediated oligonucleotide delivery 20,000 VCaP cells were seeded per well of a 96‐well culture plate and cultured for 24 h in CSS‐containing medium. Polyplexes were formed at N/P ratios of 1, 3, and 5. Incubation of cells with polyplexes was done for 30, 60, and 120 min with the final oligonucleotide concentration of 0.2 , 0.35, and 0.5 µM, using a Cy3‐AON. Next, cells were washed and cultured in CSS‐containing medium for 96 h. Incubation with PF14 alone or naked sense oligonucleotide SON‐ISE were used as controls. For splice‐correcting experiments, PF14 formulations with 0.35 µM AON‐ISE/SON‐ISE at an N/P ratio of 3 were incubated with monolayers of 22Rv1, DuCaP, or VCaP cells for 30 min followed by a washing step with fresh medium. X‐tremeGENE 9 transfected cells, untreated cells, or cells incubated with naked oligonucleotides were used as controls. At Day 4, the number of viable cells was assessed using the CellTiter‐Glo luminescence assay (Promega), following the manufacturer's instructions. Samples were transferred to a 96‐well white plate (Thermo Fisher Scientific), and luminescence was measured using a Victor3 multilabel reader (PerkinElmer). Medium only was used as background control. Each experiment was performed in three technical replicates and three biological replicates. To calculate the relative cell viability, raw luminescence unit (RLU) values for each condition were normalized to the control RLU values.

### 2.7 RNA isolation and RT‐PCR

For gene expression, 140,000 22Rv1, DuCaP, or VCaP cells were seeded per well of a 24‐well plate 1 day before the experiments and cultured in CSS‐containing medium. Four days after treatment with polyplexes or X‐tremeGENE 9 transfection, the cell culture medium was removed, and total RNA was isolated using TRIzol reagent (Invitrogen) according to the manufacturer's protocol. The concentration and purity of the RNA were determined on a Nanodrop‐1000 spectrophotometer (Thermo Scientific). Subsequently, 2 μg of total RNA was treated with DNaseI and used to synthesize complementary DNA (cDNA) using random hexamer primers and SuperScript II Reverse Transcriptase (Invitrogen). Real‐time PCR (qPCR) analysis was performed using LightCycler 480 SYBR Green I Master Mix (Roche) and primers specific for *AR‐V7* (5ʹ‐CGTCTTCGGAAATGTTATGAAGC‐3ʹ and 5ʹ‐ GAATGAGGCAAGTCAGCCTTTCT‐3ʹ), *AR‐FL* (5ʹ‐AAGGAACTCGATCGTATCATTGC‐3ʹ and 5ʹ‐TTGGGCACTTGCACAGAGAT‐3ʹ), and *HP1BP3* (5ʹ‐TGGAATATGCAATCTTGTCTGC‐3ʹ and 5ʹ‐GAACCCTTTCCCAGAGATCTG‐3ʹ). Crossing‐point (Cp) values were determined using the LightCycler 480 SW 1.5 software (Roche). Expression levels of *HP1BP3* were used for normalization, and relative gene expression levels were calculated according to the mathematical model for relative quantification in real‐time polymerase chain reaction (PCR).^13^

### 2.8 Western blot analysis

Four days after treatment with PF14 and 0.35 µM AON‐ISE/SON‐ISE formulations of an N/P ratio of 3 or after transfection of 0.35 µM AON‐ISE/SON‐ISE with X‐tremeGENE 9, 22Rv1 cells were lysed using Laemmli lysis buffer (1 mM CaCl_2_, 2% sodium dodecyl sulfate [SDS], 60 mM Tris‐Glycine pH 6.8) supplemented with 1:50 β‐mercaptoethanol (Merck). Lysates were homogenized by sheering them through a 0.5 × 25 mm syringe needle. Protein concentration was measured using the Odyssey CLx Imaging System (LI‐COR) and Image Studio software (LI‐COR), after staining with Coomassie brilliant blue (Merck) with serial dilutions of bovine serum albumin as a standard. Whole‐cell extracts were subjected to sodium dodecyl sulfate–polyacrylamide gel electrophoresis (SDS‐PAGE) using 10% polyacrylamide gels. Proteins were electrotransferred onto polyvinylidene fluoride (PVDF) membranes (Hybond 0.45 µm, Amersham Biosciences). Membranes were blocked for 1 h in phosphate buffered saline‐tween 20 (PBS‐T)/5% nonfat dry milk and incubated overnight with primary antibody. The mouse monoclonal‐antibody anti‐AR‐V7 (Precision antibody, AG10008), the rabbit polyclonal AR antibody N20 (Santa Cruz, SC‐816) and the mouse monoclonal‐antibody anti‐β‐actin (Sigma‐Aldrich, A5441) were used, diluted 1:500, 1:10,000, and 1:5000 in PBS‐T/5% nonfat dry milk, respectively. The conjugated Donkey‐anti‐Rabbit antibody (Amersham Biosciences, N4934) or sheep‐anti‐mouse antibody (Amersham Biosciences, NXA931) diluted 1:50,000 in PBS‐T were used as secondary antibodies. Protein bands were detected using Electrochemiluminescence and Hyperfilm (Amersham Biosciences). Results were reproduced in two independent experiments.

### 2.9 Confocal laser scanning microscopy

For microscopy, 35,000 DuCaP or VCaP cells were cultured per well of an 8‐well ibiTreat µ‐Slide (ibidi). Polyplexes were prepared with PF14 and Cy3‐ or AF568‐AON. Four days after transfection, cells were washed for 5 min with PBS containing 5 µg/ml Hoechst 33342 (Invitrogen; Merck) to visualize nuclei. Live‐cell confocal microscopy was performed using the Leica TCS SP8 with a temperature‐controlled stage at 37°C (Leica Microsystems), with an HCX PL APO 63 x N.A. 1.2 water immersion objective. Frame sequential images were obtained for which fluorescence was excited at 405 nm (Hoechst) and 578 nm (AF568), and emission collected between 410 and 585 nm (Hoechst) and 609–654 nm (AF568).

### 2.10 Statistical analysis

For cell viability assays with 22Rv1, DuCaP, and VCaP cells, data are presented as means ± SEM from at least three independent experiments with three technical replicates. Two‐tailed unpaired *t* tests were performed using GraphPad Prism 7 (GraphPad Software). Pearson correlation coefficients were used to determine the relationships between relative gene expression profiles, considering a 95% confidence interval. A *p*‐value of <0.05 was considered statistically significant and *p* < 0.05 is represented by one star (*) and *p* < 0.01 is represented by two stars (**).

## 3 RESULTS

### 3.1 Uptake efficiency

**Figure 1:**
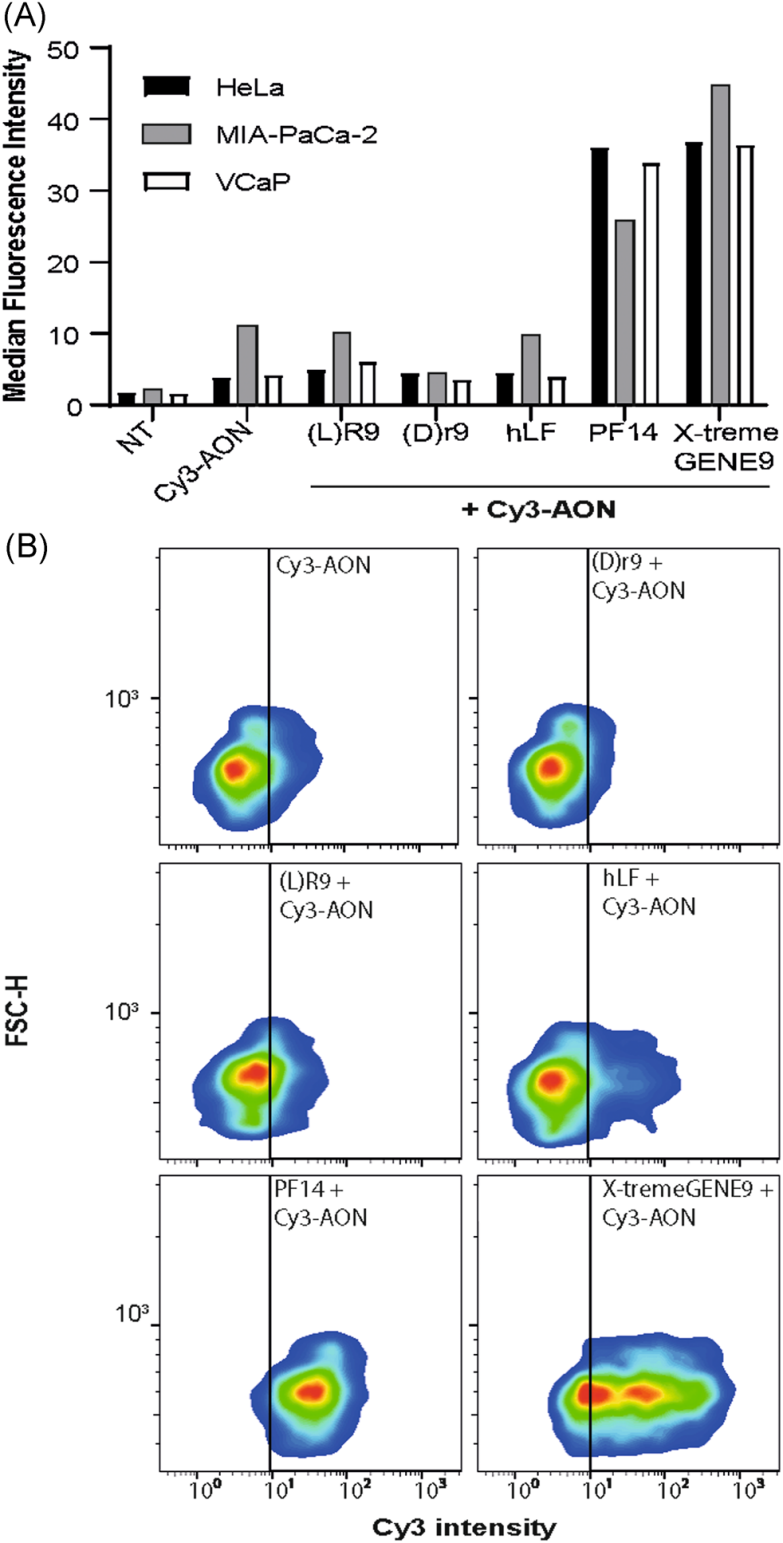
Uptake efficiency of AON‐containing polyplexes. (A) Polyplex formulations of (L)‐R9, (D)‐r9, hLF, and PF14 with 0.5 µM Cy3‐labeled AON at an N/P ratio of 5, incubated for 2 h over monolayer cultures of HeLa, MIA PaCa‐2, and VCaP cells. X‐tremeGENE 9‐mediated transfection and cells untreated or treated with naked Cy3‐labeled AON were used as controls. Graphs depict the mean fluorescent intensities. (B) Fluorescence intensity plotted against forward scatter (FSC) values of VCaP cells treated with diverse Cy3‐AON polyplex formulations and controls, as described in A. Data represents a single experimental replicate. AON, antisense oligonucleotide; hLF, human lactoferrin‐derived peptide; N/P, amino/phosphate ratio; VCaP, vertebral cancer of the prostate [Color figure can be viewed at wileyonlinelibrary.com]

Polyplexes formed with the four different CPPs, PF14, hLF, (L)‐R9, and (D)‐r9 were compared for their capacity to interact with and be internalized by the cells. For these initial experiments, a fluorescently labeled AON was used. Even though (L)‐R9 and (D)‐r9 only differ in their stereochemistry, we had shown before that the l‐peptide R9 shows superior uptake efficiency, both for the free peptide and as polyplex.^14^, ^15^ PF14 outperformed the other CPPs, mediating delivery of a Cy3‐AON in all three different cancer cell lines at levels similar to those obtained when AONs were transfected into the cells by the lipid‐based delivery agent X‐tremeGENE 9. In contrast, cells incubated with hLF, (L)‐R9, or (D)‐r9 polyplexes only showed little fluorescence, comparable to gymnotic uptake (Figure 1A). In accordance with our previous results, the d‐peptide r9 was less effective than the l‐peptide (R9). In the CRPC‐derived cell line VCaP, incubation with PF14 polyplexes resulted in a uniform population of cells positive for Cy3 fluorescence (Figure 1B). By comparison, for X‐tremeGENE 9 two populations with different uptake efficiencies were present.

### 3.2 Optimal parameters for PF14‐mediated delivery

**Figure 2:**
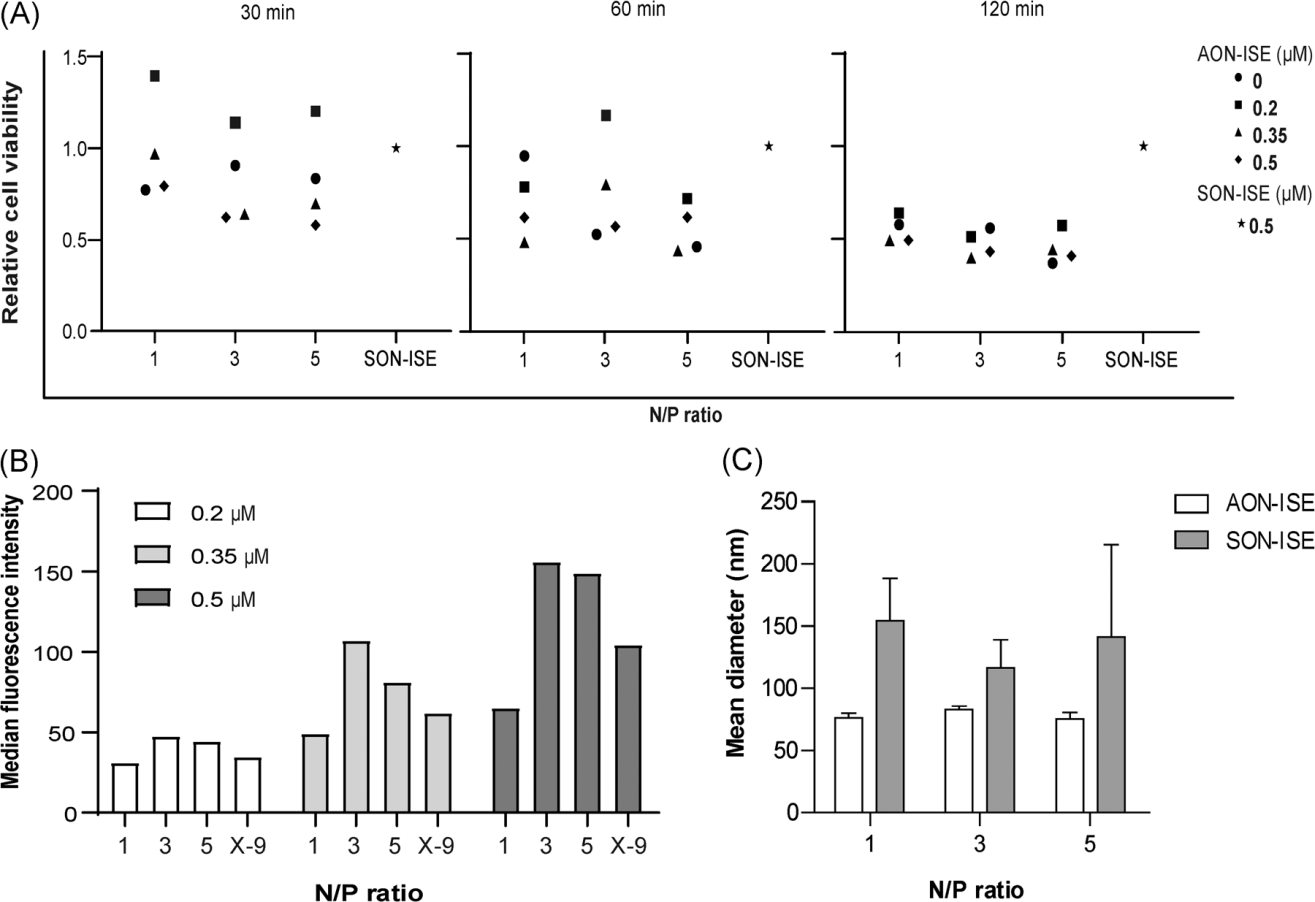
Optimal parameters for PF14‐mediated delivery of AONs. (A) Relative proliferation of VCaP cells after 30, 60, and 120 min incubation with PF14 polyplexes formulations prepared with 0.2 , 0.35 , and 0.5 µM Cy3‐labeled AON, at N/P ratios of 1, 3, and 5. PF14 alone and naked SON‐treated cells are shown as controls. Data points represent the mean of three technical replicates. (B) Cellular fluorescence for a monolayer culture of VCaP cells incubated with polyplex formulations of PF14 with Cy3‐labeled AON of 0.2, 0.35, and 0.5 µM of oligonucleotide in an N/P ratio of 1, 3, and 5, for 30 min. X‐tremeGENE 9‐mediated transfections with Cy3‐labeled AON at oligonucleotide concentrations described in B were used as controls (X‐9). The graph depicts the mean fluorescence intensities. Data represent a single experimental replicate. (C) Size determination of PF14 polyplex formulations with AON‐ISE or SON‐ISE at N/P ratios of 1, 3, and 5. Graph depicts the mean diameter (nm) of the polyplexes. Error bars represent the mean ± *SEM* of three independent experiments. AON, antisense oligonucleotide; N/P, amino/phosphate ratio; VCaP, vertebral cancer of the prostate

To determine the optimal conditions for the delivery of AONs by PF14, three different parameters were evaluated, which were the formulation N/P ratio, the concentration of oligonucleotide and the incubation time of VCaP cells with the polyplexes. After 30 and 60 min of incubation, cell viabilities varied greatly with no systematic dependence on AON concentration. Also, all N/P ratios showed a similar effect on cell viability. After 120 min incubation, there was a uniform reduction in cell viability of about 50% in comparison to the control‐treated with naked SON‐ISE, with a slight dependence of the reduction of viability on N/P ratio (Figure 2A).

Analysis of uptake efficiency at 30 min of incubation showed a dose‐dependent effect of the oligo concentration on cellular uptake for all formulation conditions. An N/P ratio of 1 was the least efficient in promoting uptake and an N/P ratio of 3 the most efficient, outperforming even X‐tremeGENE 9‐mediated transfection (Figure 2B). Of note, AON/PF14 polyplex size was hardly affected when using different N/P ratios but was surprisingly different between AON and SON (Figure 2C). Altogether, to ensure targeting of a high number of cells using a minimal quantity of peptide and oligonucleotide and to minimize toxicity, an N/P ratio of 3, an oligonucleotide concentration of 0.35 μM, and an incubation time of 30 min were determined as the most optimal parameters for testing of oligonucleotide activity.

### 3.3 Intracellular distribution of PF14 polyplexes

**Figure 3:**
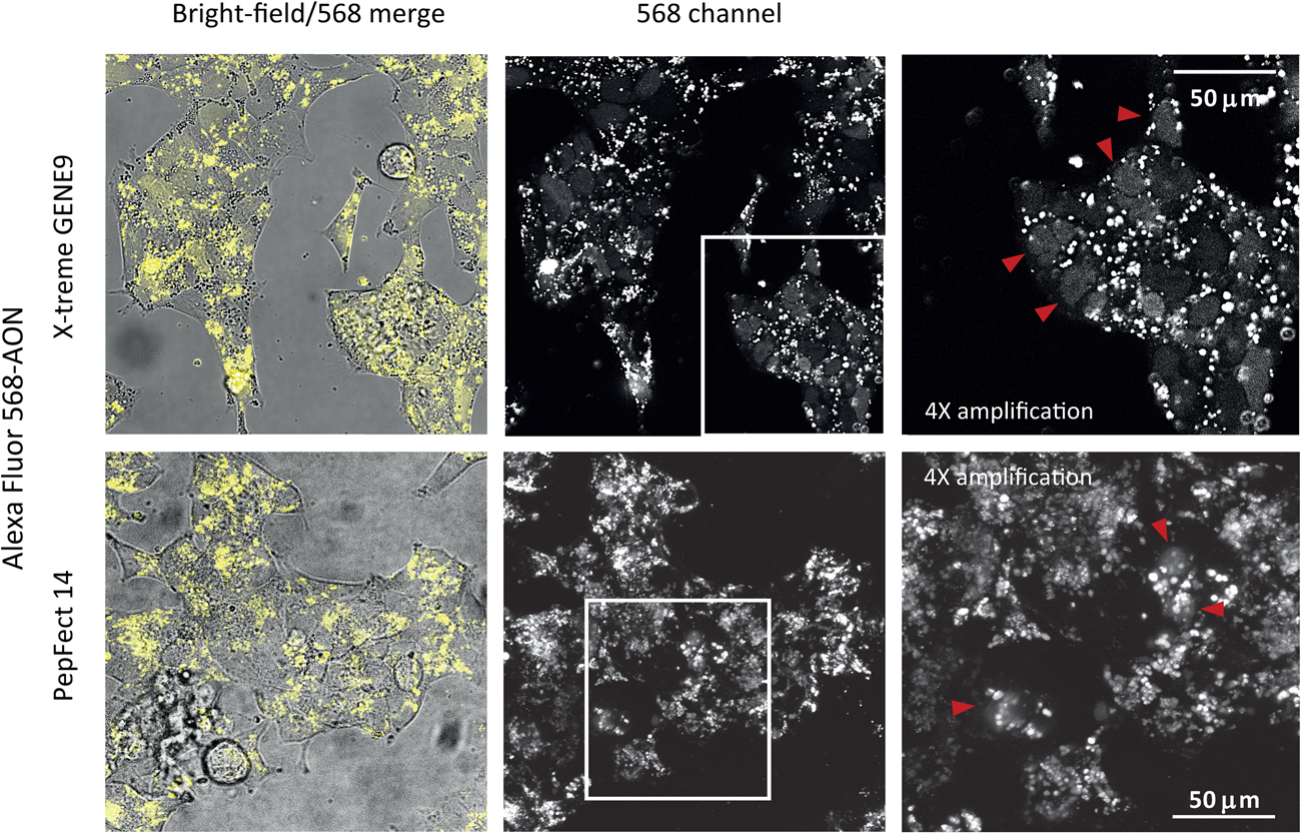
Intracellular distribution of AONs. AF568‐labeled AONs were transfected into VCaP cells using X‐tremeGENE 9 or delivered as PF14 polyplexes. Images were obtained at Day 4 posttreatment. Red arrow heads show AF568‐positive nuclei. AON, antisense oligonucleotide; VCaP, vertebral cancer of the prostate [Color figure can be viewed at wileyonlinelibrary.com]

By using an AF568‐labeled AON, we evaluated the cellular uptake and intracellular distribution of the delivered AON. In line with our flow cytometry data, both X‐tremeGENE 9 and PF14 yielded high intracellular fluorescence. In both cases, fluorescence was mostly present in large punctate structures, most likely corresponding to endosomes. For X‐tremeGENE 9 the major part of cells also showed a clearly discernible nuclear staining. By comparison, for PF14, although in fewer cells, a homogenous fluorescence inside the nuclei was also present (Figure 3). Nuclear localization of the oligonucleotides is essential for splice‐correcting AONs as they target pre‐mRNA molecules.

### 3.4 Splice‐correcting activity of PF14‐delivered AONs

**Figure 4:**
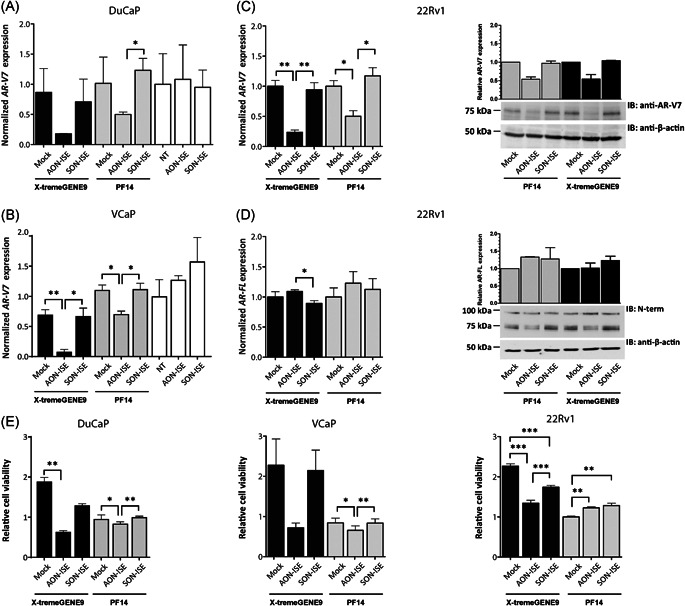
*AR* splice correction by a PF14‐formulated AON. DuCaP (A, E), VCaP (B, E) and 22Rv1 (C, D, E) cells were treated for 30 min with PF14 polyplexes formed with 0.35 µM AON‐ISE or SON‐ISE at an N/P ratio of 3. Cells untreated or treated PF14 alone, X‐tremeGENE 9 alone or with naked oligonucleotides were used as controls. (A, B, C) *AR‐V7* and *AR‐FL* mRNA were measured by RT‐qPCR analysis, 4 days after transfection with X‐tremeGENE 9 or treatment with PF14 polyplexes and normalized to *HP1BP3* mRNA expression. Error bars represent the mean ±* SEM* of three independent experiments with three technical replicates. Unpaired *t* test; *, *p* < 0.05; **, *p* < 0.01. (C, D) Western blot analysis of AR‐V7 (band around 75 kDa with anti‐ARV7 or N20) and AR‐FL (band around 100 kDa with N20) protein levels are shown. Protein levels of β‐actin (anti‐β‐actin) were used as a protein loading control. Error bars show the mean ± *SD* protein expression of two independent experiments. (E) Relative proliferation of 22Rv1, DuCaP, and VCaP cells treated as described in A‐D. Error bars represent the mean ± SEM of three independent experiments. Paired *t* test; *, *p* < 0.05; **, *p* < 0.01; ***, *p* < 0.001. AR, androgen receptor; AON, antisense oligonucleotide; DuCaP, dura mater cancer of the prostate; RT‐qPCR, real time quantitative polymerase chain reaction; VCaP, vertebral cancer of the prostate

Following a demonstration of uptake, next we were interested in assessing the activity of delivered AONs. It is well‐established that cellular uptake as such is no predictor of activity as AONs need to be released into the cytosol.^9^, ^16^, ^17^ The antisense oligonucleotide AON‐ISE, designed to target an ISE present in the *AR* pre‐mRNA and to prevent the synthesis of an *AR‐V7* transcript, was used to form polyplexes with PF14 using the optimal parameters described earlier. Next to VCaP, DuCaP cells were also used in these experiments. In both cell lines, PF14‐mediated delivery of this AON resulted in a significant reduction of *AR‐V7* levels compared with control conditions with PF14 alone or in polyplex formulations with the sense oligonucleotide SON‐ISE (Figure 4A,B). The reduction in *AR‐V7* was about 37% and 59% in comparison to a reduction of about 74% and 88% achieved by lipid‐mediated delivery of AONs in DuCaP and VCaP cells, respectively.

To validate our findings in a cell line with a different genetic background, the CRPC‐derived 22Rv1 cell line was used. The ratio of *AR‐V7* to full‐length *AR* (*AR‐FL*) mRNA levels in 22Rv1 cells is higher than in DuCaP and VCaP cells, making it an ideal model to study AR‐V7 activity.^4^ AON‐ISE treatment delivered by PF14 or using X‐tremeGENE 9 resulted in downregulation of *AR‐V7* at both mRNA and protein levels compared with control conditions with SON‐ISE, or cells treated with PF14 or the transfection reagent alone (Figure 4C). Consistent with our previous study,^4^ treatment with AON‐ISE did not reduce full‐length *AR* levels, highlighting the specificity of our AON system (Figure 4D).

Lastly, AR‐V7 is able to promote androgen‐independent cell proliferation,^18^ which is inhibited upon knockdown of this variant.^4^ Therefore, effective delivery of this AON into the nucleus should reduce the cell viability under androgen‐depleted conditions. For PF14‐mediated delivery of AON‐ISE in DuCaP and VCaP cells this was indeed the case (Figure 4E). However, the effect on 22Rv1 cells did not reach statistical significance (Figure 4E). The degree of reduction in cell viability for all PF14 polyplex conditions was lower than for X‐tremeGENE 9‐AON formulations, which may indicate that PF14 by itself exerts some degree of cell stress, even at concentrations at which no acute toxicity was observed.

## 4 DISCUSSION

CRPC is a late‐stage disease with no curative treatment. CRPC tumors often develop several mechanisms to reactivate the androgen/androgen receptor signaling axis, which make them irresponsive to AR‐targeted therapy. One of these mechanisms is the elevated expression of constitutively active androgen receptor splice variants. Variants such as AR‐V7 support androgen‐independent tumor growth and its expression can be predictive of therapy failure.^19^ Previously, we designed an AON approach to correct splicing of *AR* pre‐mRNA preventing the synthesis of an *AR‐V7* transcript.^4^ Here, we demonstrate that CPP‐mediated delivery provides a suitable approach to deliver this AON into prostate cancer cells.

In our experiments, the amphipathic CPP PF14 outperformed both nona‐arginine enantiomers (L)‐R9 and (D)‐r9,^20^, ^21^ and the hLF peptide.^10^, ^11^ These results are in line with a recent direct comparison of oligoarginine and PF14‐mediated AON uptake in myoblasts. In this system, PF14 also yielded more efficient uptake.^9^ In addition, only PF14 polyplexes achieved delivery of the AON to the nucleus. Interestingly, this nuclear localization was more pronounced than the one observed in this study.

On average, PF14 and X‐tremeGENE 9 yielded the same AON uptake efficiency. However, for X‐tremeGENE 9 two cell populations that differed in uptake efficiency were observed, while for PF14 uptake was uniform across the entire cell population with an uptake efficiency that was intermediate with respect to the two populations observed for X‐tremeGENE 9. At this point, we cannot conclude whether the higher activity observed for delivery through the latter may be attributed to the cell population showing higher uptake efficiency. Nevertheless, the presence of two populations for the lipid‐based delivery agent X‐tremeGENE 9 is in line with what we had observed for mRNA delivery.^22^

Uptake of naked (*gymnos* in Greek) phosphorothioate oligonucleotides by cells without the use of a delivery agent is a process known as gymnosis.^23^ Gymnosis has been described in different cell types in culture and offered as an alternative approach for difficult‐to‐transfect cells. In our experiments, treatment with naked AONs and SONs resulted in poor uptake and no effect on *AR‐V7* mRNA levels or cell viability. Gymnosis has been described for oligonucleotide concentrations from 2.5 to 10 µM, whereas in our experiments CPP formulations and transfection with X‐tremeGENE 9 used a maximum concentration of 0.5 µM. These findings suggests that our low oligonucleotide doses may not be sufficient for gymnotic delivery to take place and demonstrating the gain in activity that can be obtained with delivery agents.

With respect to reduction of *AR‐V7* levels and cell viability, PF14‐mediated delivery was less efficient than lipid‐based delivery. Remarkably, the difference in activity was larger for reduction in cell viability than for transcript reduction. This difference may be attributed to the fact that in spite of the absence of acute toxicity, the PF14 nanoparticles by themselves also reduced cell viability to some extent. Another possibility is that for X‐tremeGENE 9‐mediated delivery, AON activity could mostly be attributed to the subpopulation of cells showing high uptake, while PF14 achieved a lower albeit uniform effect across the entire cell population. Importantly, we showed that the higher activity of a lipid‐based formulation such as X‐tremeGENE 9 in comparison to PF14 was only present in vitro but not in vivo.^24^

Lastly, PF14 has been reported to be amenable to alterations in the charge and fatty acid moiety for augmentation of in vivo gene delivery,^25^ as well as to the addition of targeting moieties to improve tissue specificity in vivo.^26^, ^27^, ^28^ For a peptide‐based delivery agent, extension with peptide‐based targeting ligands and also modification with small molecule targeting ligands is straightforward. The prostate‐specific membrane antigen (PSMA) is a type II transmembrane glycoprotein expressed in normal prostate epithelial cells and overexpressed on nearly all prostate cancer cells,^29^, ^30^, ^31^ with the highest expression levels found in advanced stages like CRPC.^29^, ^32^, ^33^, ^34^ Interestingly, ligand binding to PSMA results in internalization. This principle has been exploited in prostate cancer diagnosis and therapy, and several PSMA ligands have been developed with some of them currently used in the clinics.^35^, ^36^ The addition of a PSMA ligand to PF14 could increase targeting specificity to the prostate and prostate cancer cells, aiding delivery of therapeutic AONs and is, therefore, a highly interesting next step.

## 5 CONCLUSIONS

Translation of therapeutic AONs from in vitro to in vivo requires cellular targeting, sufficient cellular penetration and endosomal release, and avoidance of rapid clearance by the body. In this study, we have assessed key parameters on AON formulation and cellular delivery that serve as the basis for further development of CPP‐mediated delivery of splice‐correcting AONs for targeted therapy in prostate cancer.

## CONFLICT OF INTERESTS

The authors declare that there are no conflict of interests.

## Data Availability

Data sharing not applicable to this article as no datasets were generated or analyzed during the current study.
